# RAGE mediates S100A4-induced cell motility via MAPK/ERK and hypoxia signaling and is a prognostic biomarker for human colorectal cancer metastasis

**DOI:** 10.18632/oncotarget.1908

**Published:** 2014-04-17

**Authors:** Mathias Dahlmann, Anna Okhrimenko, Patrick Marcinkowski, Marc Osterland, Pia Herrmann, Janice Smith, Claus W. Heizmann, Peter M. Schlag, Ulrike Stein

**Affiliations:** ^1^ Experimental and Clinical Research Center, Charité University Medicine Berlin and Max-Delbrück-Center for Molecular Medicine, Robert-Rössle-Straße 10, 13125 Berlin, Germany; ^2^ Max-Delbrück-Center for Molecular Medicine, Robert-Rössle-Straße 10, 13125 Berlin, Germany; ^3^ University Children's Hospital, Division of Clinical Chemistry and Biochemistry, Steinwiesstrasse 75, 8032 Zürich, Switzerland; ^4^ Charité Comprehensive Cancer Center, Charité University Medicine, Invalidenstraße 80, 10117 Berlin, Germany

**Keywords:** Colorectal cancer, metastasis, S100A4, RAGE, signaling

## Abstract

Survival of colorectal cancer patients is strongly dependent on development of distant metastases. S100A4 is a prognostic biomarker and inducer for colorectal cancer metastasis. Besides exerting intracellular functions, S100A4 is secreted extracellularly. The receptor for advanced glycation end products (RAGE) is one of its interaction partners. The impact of the S100A4-RAGE interaction for cell motility and metastasis formation in colorectal cancer has not been elucidated so far. Here we demonstrate the RAGE-dependent increase in migratory and invasive capabilities of colorectal cancer cells via binding to extracellular S100A4. We show the direct interaction of S100A4 and RAGE, leading to hyperactivated MAPK/ERK and hypoxia signaling. The S100A4-RAGE axis increased cell migration (P<0.005) and invasion (P<0.005), which was counteracted with recombinant soluble RAGE and RAGE-specific antibodies. In colorectal cancer patients, not distantly metastasized at surgery, high RAGE expression in primary tumors correlated with metachronous metastasis, reduced overall (P=0.022) and metastasis-free survival (P=0.021).

In summary, interaction of S100A4-RAGE mediates S100A4-induced colorectal cancer cell motility. RAGE by itself represents a biomarker for prognosis of colorectal cancer. Thus, therapeutic approaches targeting RAGE or intervening in S100A4-RAGE-dependent signaling early in tumor progression might represent alternative strategies restricting S100A4-induced colorectal cancer metastasis.

## INTRODUCTION

Colorectal cancer (CRC) is the third most common type of cancer worldwide, with more than 1.2 million new cases and over 600,000 deaths in the year 2008 [1]. The major cause for the high mortality of this disease is the formation of distant metastases. Patients diagnosed with CRC at an early stage have a 5-year-survival rate of about 90%, which is lowered to 65% by occurring regional lymph node metastases, and drops to less than 10% if the patient is diagnosed with distant metastases [2].

The small Ca^2+^ binding protein S100A4 has been reported to be highly expressed in different cancer tissues (reviewed in [3]). High S100A4 expression in tumors correlates with increased metastasis formation [4], which has also been shown for CRC [5,6]. In turn, the reduction of S100A4 expression in CRC-xenografted mice decreased the occurrence of metastases [7,8]. S100A4 can drive metastasis formation in several ways. Intracellular interactions with components of the cytoskeleton, as well as tumor suppressing proteins modulate the motility of cancer cells [9,10]. Additionally, the occurrence of S100A4 in the tumor-stroma microenvironment aggravates metastasis formation, partially by remodeling the extracellular matrix or the recruitment of factors of the immune system [11-13].

The receptor of advanced glycation end products (RAGE) is an interaction partner of extracellular S100A4 [14]. RAGE is a single membrane spanning protein of the immunoglobulin superfamily. Although the basal expression of RAGE is low in most tissue types, it can be up-regulated as a cellular response to pathogenic environments such as inflammatory conditions [15,16]. Systemic inflammation in the intestinal tract, caused e.g. by obesity or type 2 diabetes, is counted among the risk factors for the development of CRC [17]. The effect of RAGE expression on the progression of CRC has been shown in murine CRC models [18,19]. Indirect evidence of an elevated risk of CRC caused by RAGE-mediated signaling has been provided by epidemiological studies, focused on soluble RAGE (sRAGE) levels in CRC patients [20,21].

In this study we addressed the impact of a potential S100A4-RAGE interaction for mediating S100A4-induced cell motility and metastasis formation of CRC. Here we demonstrate the RAGE-dependent increase in the metastatic potential of CRC cells via binding to extracellular S100A4. We show the direct interaction of RAGE and rS100A4, leading to hyperactivation of the mitogen activated protein kinase (MAPK)/ extracellular signal-regulated kinase (ERK) pathway and of the hypoxia signaling pathway. Increased cellular motility of RAGE-overexpressing cells upon treatment with extracellular human recombinant S100A4 (rS100A4) protein could be reversed by RAGE-antagonists, like recombinant sRAGE (rsRAGE) and RAGE-specific antibodies. In CRC patients, high RAGE expression in primary tumors correlated with metastasis formation, as well as with shorter metastasis-free and overall survival.

## RESULTS

### Direct interaction of S100A4 and RAGE leads to enhanced cellular motility in CRC cell lines

**Fig 1:**
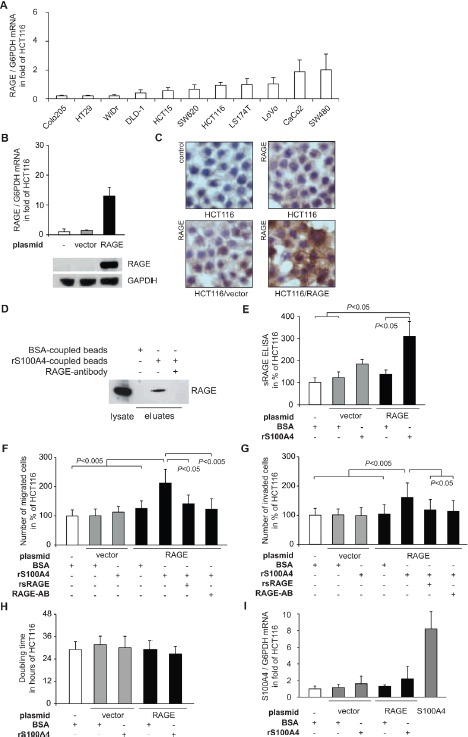
S100A4-RAGE interaction leads to release of sRAGE and increased cellular motility of the CRC cell line HCT116 A) RAGE mRNA expression levels in a panel of CRC cell lines, determined by qRT-PCR. Data represent mean RAGE/G6PDH mRNA ratios ± SD. Results were normalized to HCT116 cells. B) Generation of HCT116/RAGE cells clones. HCT116 cells were transfected with either a RAGE expression plasmid (black bars) or the empty control vector (grey bars). Data represent mean RAGE/G6PDH mRNA ratios ± SD. Results were normalized to untreated HCT116 cells. For Western blot analyses, equal loading was confirmed by GAPDH protein detection. C) Immuncytochemistry staining of HCT116, HCT116/vector, and HCT116/RAGE with RAGE-specific antibodies. Nuclei were stained with haematoxylin. D) Interaction of S100A4 and RAGE. Total lysate of HCT116/RAGE cells (lane 1) was incubated with magnetic beads, coupled with BSA (lane 2), or human rS100A4 (lanes 3,4). RAGE-specific antibody was added to an aliquot of the lysate, prior to bead incubation (lane 4). Bead eluates were blotted and incubated with anti-RAGE antibodies. E) Release of sRAGE upon rS100A4 binding. Supernatants of HCT116, HCT116/vector, and HCT116/RAGE cells were subjected to an ELISA for human sRAGE after 24 hours incubation with BSA or rS100A4. The results were normalized to untreated HCT116 cells. Data represent mean values ± SD. F,G) Cell migration (F) and cell invasion (G) of HCT116, HCT116/vector, and HCT116/RAGE after treatment with BSA or rS100A4. The interaction of S100A4 and RAGE was blocked by supplementing either rsRAGE or RAGE-specific antibody. Numbers of migrated and invaded cells were normalized to untreated HCT116 cells, respectively. Data represent mean values ± SD. H) Doubling times of HCT116, HCT116/vector and HCT116/RAGE cells were determined by MTT assays. Data represent mean values ± SD. I) S100A4 mRNA expression levels of HCT116, HCT116/vector, HCT116/RAGE, after treatment with BSA or rS100A4. HCT116/S100A4 cells serve as positive control. Data represent mean S100A4/G6PDH mRNA ratios ± SD. Results were normalized to HCT116 cells.

We first addressed the interaction of recombinant S100A4 and RAGE in CRC cells. Therefore, we analyzed the RAGE expression in 10 CRC cell lines (Fig. 1A). In order to isolate the impact of RAGE in response to rS100A4, and to mimic the up-regulation of RAGE in cancer tissue, we ectopically overexpressed RAGE in HCT116, SW620 and DLD-1 cells.HCT116/RAGE cells showed a 12-fold increase of RAGE mRNA expression, compared to the control cell lines HCT116 and HCT116/vector. RAGE overexpression was confirmed at the protein level by Western blotting (Fig. 1B) and by immunocytochemistry of RAGE in the cells (Fig. 1C).

We analyzed the receptor-ligand binding *in vitro* by precipitating RAGE with S100A4-coupled magnetic beads in HCT116/RAGE cells. We demonstrated clearly the presence of RAGE in the eluate fraction after the pull-down and thereby the physical interaction of S100A4 and RAGE. This interaction disappeared by pre-incubation of the lysate with a RAGE-specific antibody (Fig. 1D).Upon ligand binding, cells can release soluble forms of RAGE in the intercellular space which then act as soluble decoy receptors by competing with binding of ligands [22]. We measured the amount of sRAGE in the medium of the cells via RAGE-specific ELISA, recognizing N-terminal RAGE fragments. The ectopic overexpression of RAGE in HCT116/RAGE cells alone was not sufficient to release elevated amounts of sRAGE. However, when we stimulated the HCT116/RAGE with rS100A4 we found a significant accumulation of sRAGE in the medium, compared to untreated HCT116 and HCT116/vector cells (Fig. 1E).

After demonstrating the direct S100A4-RAGE interaction and a cellular response by releasing sRAGE following rS100A4 treatment, we determined the impact of RAGE activation by extracellular S100A4 for the metastatic potential of the cells. Remarkably, the number of migrated HCT116/RAGE cells significantly increased upon treatment with rS100A4 (Fig. 1F). This increase in migration could be counteracted by pre-incubation with rsRAGE. The excess of rsRAGE captures rS100A4 molecules and restricts the activation of cellular RAGE at the plasma membrane. Furthermore, treatment of the HCT116/RAGE cells with a RAGE-specific antibody prevented RAGE activation by intervening in the S100A4 binding, thereby counteracting the induction of cell migration (Fig. 1F). RAGE overexpression in HCT116/RAGE cells alone, without rS100A4 treatment, was not sufficient to induce cell migration in cell culture. Likewise, treatment of HCT116 and HCT116/vector cells with rS100A4 had also no effect on cell migration (Fig. 1F).

The ability of RAGE-overexpressing cells to invade through an additional layer of Matrigel was also significantly increased when treated with rS100A4. The incubation with rsRAGE or with the RAGE-specific antibody also resulted in decreased numbers of invaded HCT116/RAGE cells. Again, RAGE overexpression in HCT116/RAGE cells alone, without rS100A4 treatment, or treatment of HCT116 and HCT116/vector cells with rS100A4 did not result in modulated invasive abilities (Fig. 1G).

Next, we addressed the impact of S100A4-RAGE interaction for proliferation. However, when we incubated HCT116/RAGE cells with rS100A4, we did not see any significant effect on cellular growth, compared to the untreated parental HCT116 or to HCT116/vector cells, as shown by the doubling time for each subline (Fig. 1H). Application of rS100A4 did not significantly change endogenous S100A4 mRNA expression in HCT116/vector and HCT116/RAGE cells, compared to the control cell line HCT116 (Fig. 1I).

**Fig 2:**
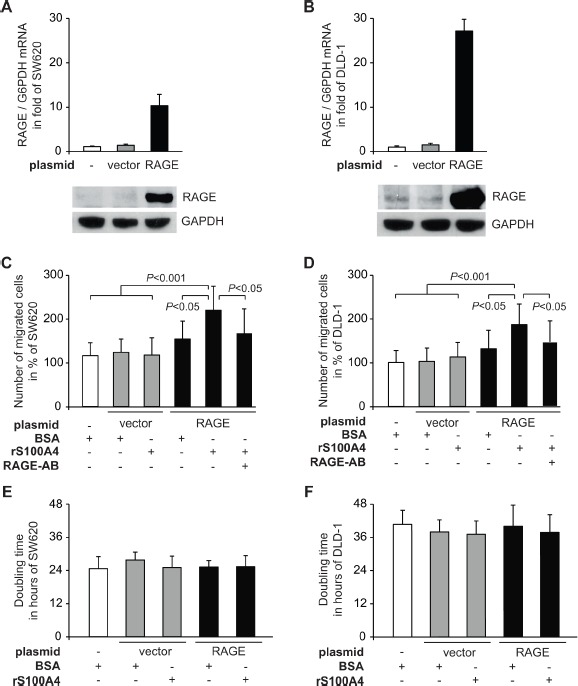
Extracellular rS100A4 increases cellular motility of the CRC cell lines SW620 and DLD-1 A,B) Generation of SW620/RAGE and DLD-1/RAGE cells clones. The CRC cell lines SW620 (A) and DLD-1 (B) were transfected with either a RAGE expression plasmid (black bars) or the empty control vector (grey bars). Data represent mean RAGE/G6PDH mRNA ratios ± SD. Results were normalized to each untreated parental cell line (SW620 and DLD-1, respectively). For Western blot analysis, equal loading was confirmed by GAPDH protein detection. C,D) Cell migration of the parental cell lines SW620 (C) and DLD-1 (D), and its derived sublines SW620/vector, SW620/RAGE, DLD-1/vector, DLD-1/RAGE after treatment with BSA, rS100A4, or with supplemented RAGE-specific antibody. Results were normalized to the parental cell lines (SW620 and DLD-1, respectively). Data represent mean values ± SD. E,F) Doubling times of the parental cell lines SW620 (E) and DLD-1 (F), and their derived cell clones expressing the empty vector or RAGE were determined by MTT assays. Data represent mean values ± SD.

The increase in cellular motility of RAGE-overexpressing cells upon treatment with rS100A4 was confirmed in additional CRC cell lines. We generated the following SW620- as well as DLD-1-derived sublines: SW620/vector and SW620/RAGE, DLD-1/vector and DLD-1/RAGE (Fig. 2A,B). Also in SW620/RAGE and DLD-1/RAGE cells, treatment with rS100A4 resulted in a significant increase in cell migration (Fig. 2C,D). Again, RAGE overexpression in SW620/RAGE and DLD-1/RAGE cells alone, without rS100A4 treatment, was not sufficient to induce the migration rate, and rS100A4 treatment of the vector-transfectants SW620/vector and DLD-1/vector did not lead to elevated numbers of migrated cells. Proliferation of SW620 and DLD-1 cells was not affected, neither by RAGE overexpression nor by incubation with rS100A4 (Fig. 2E,F).

### Extracellular S100A4 induces RAGE-mediated hyperactivation of MAPK/ERK and hypoxia signaling

**Fig 3:**
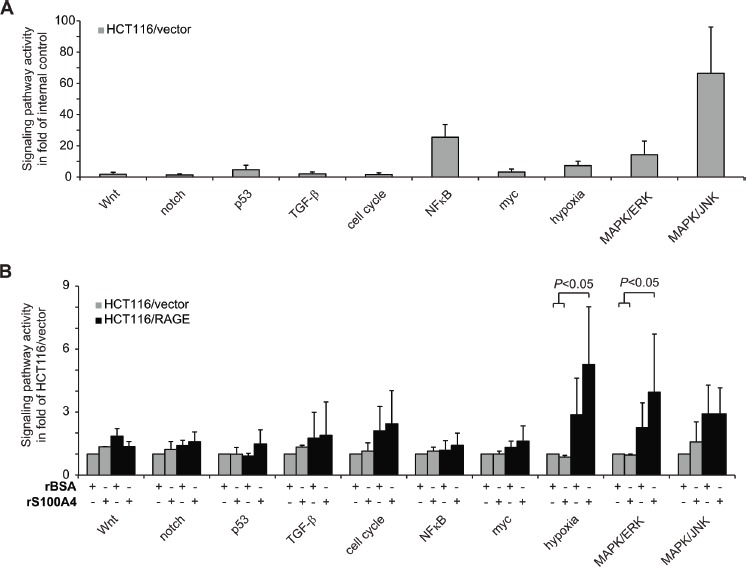
S100A4-RAGE interaction hyperactivates MAPK/ERK and hypoxia signaling pathways A) Relative luciferase activity of HCT116/vector cells, transfected with signaling pathway specific reporter constructs. Results were normalized to the internal control. Data represent mean values ± SD. B) Relative luciferase activity of HCT116/vector (grey bars) and HCT116/RAGE cells (black bars), transfected with signaling pathway specific reporter constructs. Cells were incubated for 24 h with BSA or rS100A4. Results were normalized to the respective untreated HCT116/vector cells. Data represent mean values ± SD.

To identify the signaling pathways involved in the RAGE-dependent increase of cellular motility upon rS100A4 treatment, we determined the activity of a panel of cancer-related signaling pathways in HCT116/RAGE cells. First we analyzed the basal activity of different cancer-related signaling pathways in untreated HCT116/vector cells. We observed elevated signal intensities of the reporter constructs for MAPK/ERK- and MAPK/JNK-driven transcription, as well as for NFκB- and hypoxia-pathway activities (Fig. 3A). We then normalized the pathway-dependent signals to the untreated HCT116/vector cells and analyzed the pathways activities in RAGE-overexpressing cells and following treatment with rS100A4 (Fig. 3B). Overexpression of RAGE in HCT116/RAGE cells alone resulted in elevated reporter activities of the MAPK/ERK and MAPK/JNK pathways as well as of the hypoxia signaling pathway. In addition, the stimulation of HCT116/RAGE cells with rS100A4 induced a significant hyperactivation of the MAPK/ERK and the hypoxia signaling pathways, but no further increase in the MAPK/JNK pathway reporter signals (Fig. 3B). Treatment of HCT116/vector cells with rS100A4 did not affect pathway activities significantly.

**Fig 4:**
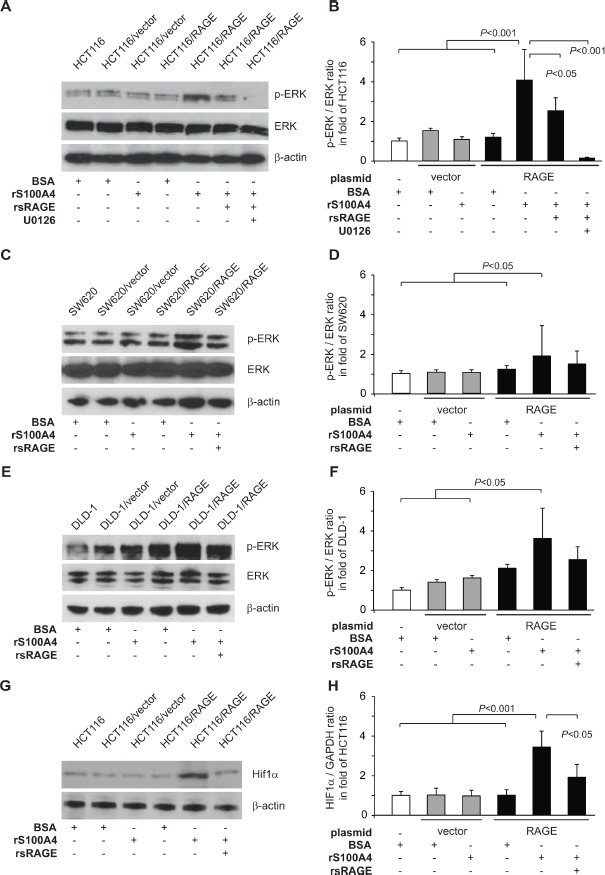
S100A4-RAGE interaction increases ERK phosphorylation and Hif1α protein level A-F) Increase in ERK phosphorylation by S100A4-RAGE interaction. Western blot analysis of ERK phosphorylation in HCT116, HCT116/vector, and HCT116/RAGE cells (A), in SW620, SW620/vector, and SW620/RAGE cells (C), and in DLD-1, DLD-1/vector, and DLD-1/RAGE cells (C) after incubation for one hour with BSA (lanes 1,2,4), rS100A4 (lanes 3,5,6,7), additional treatment with rsRAGE (lane 6), or treatment with MEK inhibitor U0126 (lane 7). Membranes were incubated with antibodies against ERK, p-ERK, and β-actin. Intensities of p-ERK signals for each cell line (B,D,F, respectively) were quantified and normalized to its respective total ERK signals. G,H) Increase in Hif1α protein level. Western blot analysis of Hif1α in HCT116, HCT116/vector and HCT116/RAGE cells after incubation for 24 hours with BSA (G; lanes 1,2,4), rS100A4 (G; lanes 3,5,6), or additional treatment with rsRAGE (lane 6). Membranes were stained with antibodies against Hif1α and β-actin. Intensities of Hif1α protein signals were quantified and normalized to the respective β-actin signals (H).

Next, we focused on the rS100A4-induced activation of the MAPK/ERK signaling pathway in HCT116/RAGE cells. Treatment with rS100A4 increased the amount of phosphorylated ERK (p-ERK) in these RAGE-overexpressing cells, compared to the controls, thereby confirming the activation of MAPK/ERK pathway. Parallel incubation with rsRAGE reduced the phosphorylation of ERK, whereas the total amounts of ERK remained unchanged (Fig. 4A,B). Preincubation of the cells with the MEK inhibitor U0126 resulted in only minimal residual ERK-phoshorylation (Fig. 4A,B).

The increase in p-ERK by treatment with extracellular rS100A4 was confirmed in other RAGE-overexpressing CRC cells. Incubation with rS100A4 led to increased amounts of p-ERK in SW620/RAGE cells, but not in the control cell lines SW620 and SW620/vector (Fig. 4C,D). Simultaneous treatment with rsRAGE reduced p-ERK levels in SW620/RAGE cells. Same observations were made for DLD-1/RAGE cells, which did react with elevated p-ERK levels to rS100A4 incubation. Again, treatment with rsRAGE lowered the amount of p-ERK. DLD-1 and DLD-1/vector cells were not affected by any kind of treatment (Fig. 4E,F).

Validation of the hypoxia signaling pathway activation upon rS100A4 incubation was performed by quantifying the amount of endogenous Hif1α protein, the major regulator of hypoxia response. Treatment of HCT116/RAGE cells with rS100A4 led to an increased protein level of Hif1α in cell lysates, that could be reduced by simultaneous treatment with rsRAGE (Fig. 4G,H). Neither RAGE-overexpression alone not rS100A4 incubation resulted in modulated Hif1α levels in the control cells.

### RAGE expression in primary colorectal tumors correlates with patient survival

We examined the expression of RAGE in tumor samples of a cohort of 60 patients with CRC in stages I, II, and III (without distant metastases at the day of tumor resection [23]) by gene-specific quantitative RT-PCR (Table 1). We first addressed the question on whether RAGE expression levels correlate with the development of metachronous metastases.

**Fig 5:**
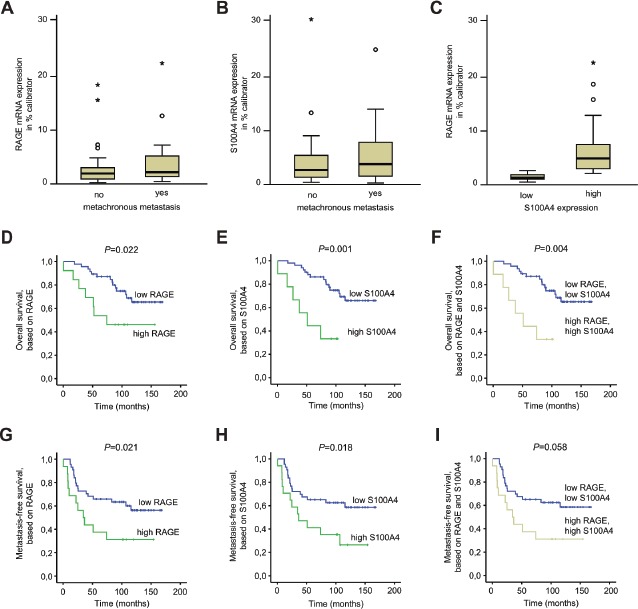
RAGE expression in primary colorectal tumors correlates with patient survival A-C) RAGE (A) and S100A4 (B) mRNA expression is higher in primary CRCs, that metachronously develop distant metastases. RAGE mRNA expression is higher in S100A4 high expressing tumors (c; P=0.001). D-I) Overall survival (D-F) and metastasis-free survival (G-I) of CRC patients, based on RAGE (D,G), S100A4 (E,H) and combined RAGE and S100A4 (F,I) expression. RAGE as well as S100A4 mRNA expression levels were determined by qRT-PCR in microdissected tumor cell populations of primary, not yet metastasized tumors of stages I, II and III. The cut-off values to distinguish low and high expression levels were determined by ROC analyses.

Metastases were more frequently formed in patients with high RAGE expression in their primary tumors (metachronously metastasized: RAGE mRNA expression/% calibrator, median 2.119) compared to those who did not metastasize in a follow-up of 12 years (non-metastasized: RAGE mRNA expression/% calibrator; median 1.888) (Fig. 5A). Likewise, high S100A4 expression also correlated to the metastases development (metastasized: S100A4 mRNA expression/% calibrator, median 3.715 vs. non-metastasized: S100A4 mRNA expression/% calibrator, median 2.344) (Fig. 5B). Furthermore tumors with high S100A4 expression also expressed RAGE at high levels (P<0.001, Fig. 5C).

We then linked RAGE expression levels to patient survival using Kaplan-Meier analysis. The cut-off was determined by ROC analyses. We found a significant correlation of elevated RAGE expression with decreased overall survival (OS) (P=0.022; Fig. 5D) as well as decreased metastasis-free survival (MFS) (P=0.021; Fig. 5G). The 5-year OS was 87.2% (± 4.5%) for RAGE low-expressers but 53.8% (± 13.8%) for patients with elevated RAGE expression in the tumor. High RAGE levels also correlated with shorter 5-year MFS of 37.5% (± 12.1%) compared to 65.9% (± 7.1%) for RAGE low expressing patients.

Simultaneously, we determined the S100A4 expression in the same samples. Elevated expression of S100A4 also showed a correlation with shorter OS (P=0.001; Fig. 5E) and with shorter MFS (P=0.018; Fig. 5H). Increased expression of S100A4 in tumors was also connected to shorter 5-year OS (44.4% (± 16.6%) compared to 86.2% (± 4.8%) for S100A4 low expressers) and to shorter 5-year MFS (41.2% (± 11.9%) compared to 65.1% (± 7.3%) for S100A4 low expressers).

By combining RAGE and S100A4, we also observed a significant decrease of the OS rate (P=0.004; Fig. 5F) and a strong reduction of the MFS rate when both markers genes were upregulated (P=0.058; Fig. 5I). However, the combination of RAGE and S100A4 did not improve the prognosis based on S100A4 exclusively. The 5-year OS was 44.4% (± 16.6%) for patients with elevated RAGE and S100A4 expression in their tumors compared to 87.2% (± 4.9%) for patients with low expressions of both genes. Likewise, the 5-year MFS was 37.5% (± 12.1%) when RAGE and S100A4 were simultaneously highly expressed compared to 65.1% (± 7.3%) for patients with both gene expressed at low levels.

Taken together, RAGE was identified as biomarker for disease prognosis of CRC. Based on our findings on the importance of the S100A4-RAGE interaction, therapeutic approaches targeting RAGE or intervening in RAGE dependent signaling early in tumor progression might represent alternative strategies restricting the S100A4-induced metastasis in CRC.

## DISCUSSION

Here we report the interaction of extracellular S100A4 and RAGE in CRC cells leading to hyperactivation of the MAPK/ERK and the hypoxia signaling pathway. An increase in cellular motility, after treatment with rS100A4, could be counteracted using RAGE-antagonists. Analysis of primary colorectal tumors unveiled RAGE as a biomarker for prognosis of CRC. Elevated RAGE as well as S100A4 expression levels correlated significantly with shorter MFS and OS.

S100A4 expression in cancer affects many disease related cellular responses, like angiogenesis, cell motility, invasion, and cell survival, contributing to tumor progression and metastasis formation [3]. It is a target gene of T-cell factor/β-catenin transcription complex, which is often deregulated in CRC [6,24]. Clinical data acknowledged S100A4 as a prognostic biomarker for CRC metastasis [25]. The quantification of S100A4 mRNA in plasma samples is suited as a diagnostic tool for CRC metastasis [26], which allows early therapeutic interventions to reduce S100A4 in tumor tissues, and in turn disease progression. One therapeutic aim is to target S100A4 gene expression within the tumor cells. *In vivo* application of small molecules as transcription inhibitor, or the systemic knock-down of S100A4 expression by RNAi, were successful to reduce CRC metastasis [7,8,27,28]. Other studies evaluate the inhibition of S100A4 protein function with S100A4-specific antibodies, or by blocking its binding to RAGE with antagonistic peptides, shown for glioma and pancreatic cancer [13, 29, 30]. For CRC, elevated RAGE expression in the intestine promotes tumor formation and disease progression [31,32]. However, the impact of RAGE expression on S100A4-induced cell motility and metastasis formation in CRC has not been elucidated so far.

Here we demonstrate increased cellular motility by the stimulation of RAGE-overexpressing CRC cells with rS100A4. This effect was reversed by inhibiting the S100A4-RAGE interaction with simultaneous application of RAGE antagonists.

The interaction of S100A4 and RAGE has been shown first in human articular chondrocytes by immunoprecipitation [14], and was later classified as a direct interaction by biophysical characterization of receptor-ligand binding [33]. In our study, we report the direct binding both proteins by precipitating RAGE with rS100A4, covalently bound to beads. We also provide data supporting the S100A4-RAGE interplay, by measuring the release of sRAGE into the medium, after treatment with rS100A4. Raucci and colleagues describe the release of soluble forms of RAGE upon ligand binding [22]. One mechanism involves alternative splicing of the RAGE transcript and the release of a C-terminally altered RAGE [34]. In a different mechanism, the receptor is cleaved near the transmembrane region by metalloproteases, and the extracellular ligand binding site of the receptor is released from the cell surface [22]. In both mechanisms the increased amount of soluble RAGE fragments in the intercellular space can act as soluble decoy receptors by competing with binding of ligands.

By demonstrating the binding of S100A4 to RAGE and its effect on cellular motility of CRC cell lines, we were interested in the signaling pathways involved. When we analyzed a panel of cancer related signaling pathways in the cell line HCT116/RAGE, we found a significant increase in ERK as well as hypoxia pathway activity upon treatment with rS100A4. The activation of ERK signaling in CRC cells was previously described for other RAGE ligands besides S100A4; for AGEs in Caco-2 [35], for S100P in SW480 [36], and for S100A8/A9 in MC38 and Caco-2 [31]. Interestingly, we already observed a minor increase in ERK-signaling in DLD-1/RAGE cells without rS100A4 treatment. Since these cells are kept in DMEM containing higher glucose levels, RAGE activation might have partially occurred due to increased formation of AGEs in the medium. Other groups report RAGE-independent ERK activation by extracellular S100A4 [37,38]. To the best of our knowledge, the modulation of ERK signaling in CRC cells upon S100A4-RAGE interaction has not been described before.

We also found an increase of the hypoxia signaling pathway in RAGE overexpressing cells, which was significantly enhanced by adding rS100A4. In general, an elevated Hif1α activity leads to cell type specific changes in gene expression of cell lines and cancer tissues [39]. For CRC, Hif1α was described to enhance resistance to apoptosis, increase cellular motility, and decrease response to chemotherapy [40-42]. A connection of RAGE and hypoxia response has been reported in breast and cervix carcinoma cell lines [43]. Hypoxic conditions in those cells induced the expression of RAGE and increased the RAGE-dependent signaling, as well as cellular motility. Here, we observed an increase of a Hif1α-dependent reporter activity in RAGE-overexpressing CRC cells, upon stimulation with rS100A4, verified by Western blotting. The elevated Hif1α protein level, caused by the incubation with rS100A4, was decreased when we interrupted the protein binding with rsRAGE or the RAGE-specific antibody.

In prostate cancer, recent mouse experiments revealed enhanced tumorigenesis of extracellular S100A4-RAGE interaction, by activating the NFκB signaling pathway [44]. In contrast, NFκB signaling has been shown to be constitutively active in most CRC cell lines [45]. We did not observe an increase in the NFκB pathway activity in our cells, neither by overexpressing RAGE nor by treatment with rS100A4. This is in line with a report by Boye and colleagues, demonstrating no increased NFκB pathway activation in CRC cell lines by applying rS100A4 [46]. Interestingly, in osteosarcoma cell lines, S100A4-mediated NFκB activation occurred independently of RAGE [46,47], indicating further receptors or receptor complexes interacting with S100A4. Taken together, the interaction of RAGE with rS100A4 hyperactivates MAPK/ERK and hypoxia signaling pathways. Increased migration and invasiveness of CRC cells upon rS100A4 treatment was reversed by interfering with its binding to RAGE.

It has been shown, that RAGE is up-regulated in the intestine as a result of chronic inflammation or diabetes, and leads to increased adenoma formation [32,48]. In CRC, elevated RAGE expression can form the basis for tumor formation and progression [49]. The switch to malignancy depends on the presence of RAGE ligands. The elevated expression and secretion of members of the S100 protein family into the tumor microenvironment drives the progression of the tumor and, eventually, metastasis formation [50], directly linked to patient survival. After determining the expression levels of RAGE and S100A4 in a panel of 60 human colorectal tumors, diagnosed and resected at stages I, II and III, we found a significant correlation of low overall and metastasis-free survival with high RAGE expression as well as high S100A4 expression in the tumors. However, the combination of both RAGE and S100A4 expression did not improve the survival prognosis of the patients. Ligand binding of RAGE occurs in the extracellular tumor microenvironment, with ligands secreted either by the cancer cell itself, or by the surrounding tissue [51]. Thus, a tumor with high RAGE expression does not necessarily need high endogenous S100A4 expression levels to activate the receptor.

In summary, we demonstrated the importance of the interplay of S100A4 and RAGE for hyperactivation of ERK and hypoxia signaling, correlated with increased motility of CRC cells. Furthermore, high RAGE expression in primary CRC correlates with reduced metastasis-free and overall survival of patients. Therefore, therapeutic approaches targeting RAGE or intervening in RAGE-dependent signaling early in tumor progression might represent alternative strategies restricting the S100A4-induced metastasis in CRC.

## METHODS

### Subjects and tissues

We obtained tissue specimens from 60 patients suffering from CRC with informed written consent (approved by Charité Ethics Committee, Charité University Medicine, Berlin, Germany). All patients were staged I, II, or III (not distantly metastasized at the time point of surgery). They were previously untreated, did not develop tumors of a second entity, and showed no familial history of CRC. The patients' tumors were surgically resected R0 (complete resection with no microscopic residual tumor). Twenty three patients formed distant metastases metachronously (after surgery), whereas 37 patients remained free of metastases. A comprehensive description of the analyzed tissues can be found in [23]. Tumor specimens (all were adenocarcinomas) were snap frozen in liquid nitrogen. Serial consecutive cryosections were evaluated by a pathologist and tumor cell populations were microdissected.

### Plasmid construction, cell culture, and transfection

The cDNA of human full length RAGE (OriGene) was cloned into pcDNA3.1 (Invitrogen), under the control of the CMV promoter, resulting in the plasmid pcDNA3.1/RAGE. Transfection of the cell lines HCT116, SW620 and DLD-1 with pcDNA3.1/RAGE or the empty vector resulted in the cell lines HCT116/vector, HCT116/RAGE, SW620/vector, SW620/RAGE, DLD-1/vector, and DLD-1/RAGE, respectively. All transfections were performed with Fugene HD (Promega), according to the manufacturer's instructions. Transfected cells were selected with 1 mg/ml neomycin (PAA Laboratories). All cell lines derived from HCT116 and SW620 were grown in RPMI-1640 medium, cell lines derived from DLD-1 were grown in DMEM (PAA Laboratories), each medium supplemented with 10% FCS (Invitrogen), in a humidified incubator at 37°C and 5% CO_2_. The cell lines were tested with the MycoAlert Mycoplasma Detection Kit (Lonza), and found to be free of mycoplasma. The genotype of the parental cell lines HCT116, SW620, and DLD-1 were confirmed by short tandem repeat (STR) genotyping at the DSMZ (German Collection of Microorganisms and Cell Cultures; Braunschweig, Germany).

### Real-time Quantitative Reverse Transcriptase Polymerase Chain Reaction (qRT-PCR)

Total RNA from cell culture or microdissected tumor tissues was isolated using the Universal RNA Purification Kit (Roboklon), according to the manufacturer's instructions. Quantification of RNA concentration was performed with Nanodrop (Peqlab), and 50 ng total RNA was reversely transcribed with random hexamer primers in a reaction mix of 10 mM MgCl_2_, 1 × PCR-buffer II, 250 µM pooled dNTPs, 1 U/µL RNAse inhibitor, 2.5 U/µL MuLV reverse transcriptase (Invitrogen). The reaction occurred at 42°C for 15 minutes, 95°C for 5 minutes, and subsequent cooling at 4°C for 5 minutes. The cDNA product was amplified in a total volume of 10 µl in 96-well plates using the LightCycler 480 (Roche) and the following PCR conditions: 95°C for 10 minutes, followed by 45 cycles of 95°C for 10 seconds, 60°C for 30 seconds, and 72°C for 4 seconds. For S100A4 cDNA quantification, the following primers and probes were used for a 124 bp amplicon: forward primer, 5′-gagctgcccagcttcttg-3′; reverse primer, 5′-tgcaggacaggaagacacag-3′; fluorescein isothiocyanate (FITC) probe, 5′-tgatgagcaacttggacagcaaca-3′; and LCRed640-probe, 5′-gacaacgaggtggacttccaagagt-3′ [6]. For RAGE cDNA quantification, the following primers and probes were used to amplify a 226 bp fragment: forward primer, 5′-gtcggagctaatggtgac-3′; reverse primer, 5′-ctgggcagggacttcac-3′; fluorescein isothiocyanate (FITC) probe, 5′-tggagccagaaggtggagcagta-3′; and LCRed640-probe, 5′-ctcctggtggaaccgtaaccct-3′. For cDNA quantification of the housekeeping gene glucose-6-phosphate dehydrogenase (G6PDH) the LightCycler-h-G6PDH Housekeeping Gene Set (Roche) was used, according to manufacturer's instructions, leading to a 113 bp amplicon. Mean values were calculated from duplicate qRT-PCR reactions. Each mean value of the expressed gene was normalized to the respective mean value of the housekeeping gene cDNA. The cDNA for the calibrator and the in-run standard derived from HCT116 cells was employed in serial dilutions simultaneously in each run.

### Protein extraction and Western blot

For total protein extraction, cells were lysed with RIPA buffer (50 mM Tris-HCl, 150 mM NaCl, 1% Nonidet P-40, supplemented with complete protease inhibitor tablets; Roche) for 30 minutes on ice. Protein concentration was quantified with Coomassie Plus (Bradford) Protein Assay Reagent (Pierce), according to manufacturer's instructions, and lysates of equal protein concentration were separated with SDS-PAGE and transferred to Hybond-C Extra nitrocellulose membrane (GE Healthcare). Membranes were incubated in blocking solution containing 5% nonfat dry milk at room temperature. Incubation with the appropriate primary antibody was performed overnight at 4°C, followed by incubation with the respective HRP-conjugated secondary antibody, diluted 1:10,000 in 5% BSA/TBST for 1 hour at room temperature. For RAGE detection we used the mouse anti-human RAGE A11 antibody (1:1000; sc-80652, Santa Cruz Biotechnology), for GAPDH we used goat anti-GAPDH V-18 (1:500; sc-20357, Santa Cruz Biotechnology). ERK was detected by rabbit anti-p42/44 antibodies (1:1000; #9102, Cell Signaling) and phosphorylated ERK was detected by the rabbit anti-P-p42/44 antibodies (1:1000; #9101, Cell Signaling). For Hif1α we used the rabbit anti-Hif1α antibody (1:1000; GTX127309P, GeneTex), and for β-actin we used the mouse anti-β-actin AC-15 antibody (1:10,000; A1978, Sigma-Aldrich). Antibody-protein complexes were visualized with electrochemical-luminescence reagent (100 mM Tris/HCl, 0.025% w/v luminol, 0.011% w/v para-hydroxycoumaric acid, 10% v/v dimethylsulfoxide, 0.004% v/v H_2_O_2_, pH 8.6) and subsequent exposure to CL-XPosure™ Films (Pierce). Immunoblotting for GAPDH served as protein loading control. The signals were quantified with ImageJ version 1.74f [52]. All experiments were performed at least three independent times.

### Immunocytochemistry

Cells were cultured on double-chamber slides (Nunc) and were fixated for 15 minutes with 0.04% glutaraldehyde. Endogenous peroxidase was inactivated for 20 minutes with 0.9% H_2_O_2_ and cells were permeabilized for 10 minutes with 0.5% Triton X100 in 2.5% BSA. After blocking for 1 hour with 5% BSA, cells were incubated for 2 hours with the RAGE specific antibody (1:400; sc-80652, Santa Cruz Biotechnology). Detection was performed using the biotin-based ABC kit (Dako) with diaminobenzidine as substrate, according to manufacturer's instructions. Positive signals resulted in brown staining. The slides were counterstained with haematoxylin and analyzed using a light microscope Axioplan2 (Leica) at a magnification of 40x. Negative control experiments were carried out by omitting the primary antibody.

### RAGE pull-down

Dynabeads® (Antibody Coupling Kit 143.11D; Invitrogen), were coupled with recombinant human S100A4 (kind gift of K. Boye, Oslo; [46]) or BSA, respectively, according to the manufacturers' protocol and finally blocked with 2% BSA in PBS. Three × 10^7^ HCT116/RAGE cells were lysed in RIPA buffer and incubated with 100 µl of coupled Dynabeads, with or without the presence of 25 µg RAGE H300 antibody (sc-5563, Santa Cruz Biotechnology) overnight at 4°C. The beads were washed three times with PBS, subjected to SDS-PAGE, and Western blotting was performed for detection of RAGE. The experiments were performed two times.

### Treatment with recombinant human S100A4 or blocking substances

Cells were seeded into 6-well plates up to 70% confluency and were incubated overnight in growth medium, supplemented with 10% FCS. The medium was removed and replaced with growth medium containing 0.5% FCS. The cells were either treated with rS100A4 (R&D Systems) or, as control, with BSA. Counter treatment was performed with either purified human rsRAGE, or a purified RAGE-specific antibody (both described in [53]). The purity of the proteins used for treatment (rS100A4 and rsRAGE) was analysed via SDS-PAGE and Coomassie staining (Fig. S1). The MEK inhibitor U0126 (#9903; Cell Signaling) was added to the cells at 10 µM, 1 hour before the treatment with rS100A4.

### RAGE ELISA

The quantification of sRAGE in the medium of treated and untreated cells was performed with an ELISA Kit for human sRAGE (BioVendor) according to the manufacturers' protocol. In brief, 5 × 10^5^ HCT116, HCT116/vector, and HCT116/RAGE cells were treated for 24 hours with 5 µg/ml rS100A4 or, as control, with 5 µg/ml BSA. The growth medium was removed and the concentration of sRAGE was measured. The experiment was repeated twice.

### Boyden chamber transwell migration and invasion assay

The cell lines HCT116, SW620, and DLD-1, and the respective clones, HCT116/vector, HCT116/RAGE, SW620/vector, SW620/RAGE, DLD-1/vector and DLD-1/RAGE, were used in cell migration and invasion analyses performed by Boyden chamber assays. Cells were treated for 24 hours with 5 µg/ml rS100A4 or, as control with 5 µg/ml BSA. The interaction of RAGE and S100A4 was blocked by adding either 30 µg/ml rsRAGE or 150 µg/ml purified RAGE antibody. For determining cell migration, 2.5 × 10^5^ cells were seeded into each transwell chamber with filter membranes of 12 μm pore size (Millipore). For invasion, filter membranes were coated with Matrigel (BD Biosciences; diluted 1:3 in growth medium) 4 hours before seeding 5 × 10^5^ cells, and fresh medium was added to the bottom chamber. After 24 hours (migration) or 72 hours (invasion), respectively, inserts were removed and cells which had migrated through the membrane to the lower chamber were trypsinized and counted in a Neubauer chamber (LO-Laboroptik). Each well was counted ten times. Each migration or invasion experiment was performed in duplicate. The average number of migrated or invaded cells was determined from at least three independent experiments.

### Monitoring cell proliferation by MTT

Two × 10^3^ cells of HCT116, HCT116/vector, HCT116/RAGE, SW620, SW620/vector, SW620/RAGE, DLD-1, DLD-1/vector and DLD-1/RAGE cells were seeded in 96-well plates (for each day one plate) and incubated for 24 hours to allow the cells to attach to the bottom of the wells. Determination of viable cells was performed by adding 3-(4,5-dimethyl-2-thiazol)-2,5-diphenyl-2H-tetrazolium bromide (MTT; Sigma-Aldrich) to a final concentration of 0.5 mg/ml. After 2 hours incubation at 37°C and 5% CO_2_ in a humidified incubator, the formazan crystals were resolved by 10% SDS in 10 mM HCl and the absorption was measured at 560 nm. MTT measurements were performed daily for 5 consecutive days, as triplicates in three independent experiments. The absorption data were used to calculate the doubling time of each cell line (V. Roth 2006; wwww.doubling-time.com). The growth rate of each cell line has been determined three times.

### Signaling pathway analysis

The analysis of activated signaling pathways by ectopic RAGE expression, with or without treatment with rS100A4, was performed with the Cignal Finder Cancer 10-pathway Reporter Luciferase Kit (SABiosciences), according to the manufacturers' instructions. In brief, 2 × 10^4^ cells were reversely transfected in the reporter plates and incubated over night at 37°C in a humidified incubator, followed by treatment with 5 µg/ml rS100A4 or 5 µg/ml BSA. Cells were lysed after 24 hours of incubation. The measuring of firefly luciferase and renilla luciferase activity was performed with an ‘infinity2000’ plate reader (Tecan), using StopGlo reaction mix (Promega). The pathway dependent reporter signal intensities of firefly luciferase were normalized to pathway independent renilla luciferase signal intensities. The experiment has been repeated twice.

### Statistical analysis

Statistical analyses were performed with Sigma Stat Version 3.5. Comparison of more than two groups was performed by one-way analysis of variance (ANOVA) and Bonferroni post hoc multiple comparison, or one-way ANOVA on ranks and Tukey post hoc multiple comparison, if the normality test of the data failed. *P* values less than 0.05 were defined as statistically significant. Statistical evaluation of human CRC samples was performed using the non-parametric two-sided Mann-Whitney Rank Sum test. Survival rates were calculated with Kaplan-Meier-Estimator. The cut-offs to distinguish low and high expression levels were determined using Reciever-operator-characteristics (ROC) analysis by taking the value with the highest Youden-Index. All computations were made using IBM SPSS Statistics 21.

## SUPPLEMENTARY MATERIAL


