# Supplementary material for: RAGE mediates S100A4-induced cell motility via MAPK/ERK and hypoxia signaling and is a prognostic biomarker for human colorectal cancer metastasis
Source: Oncotarget. 2014 Apr 17;5(10):3220–33. doi: 10.18632/oncotarget.1908 (PMC4102805; doi:10.18632/oncotarget.1908)
Supplement: Supplementary file 1 [file oncotarget-05-3220-s001.pdf]

RAGE mediates S100A4-induced cell motility via MAPK/ERK and hypoxia signaling and is a prognostic biomarker for human colorectal cancer metastasis

Supplementary Material

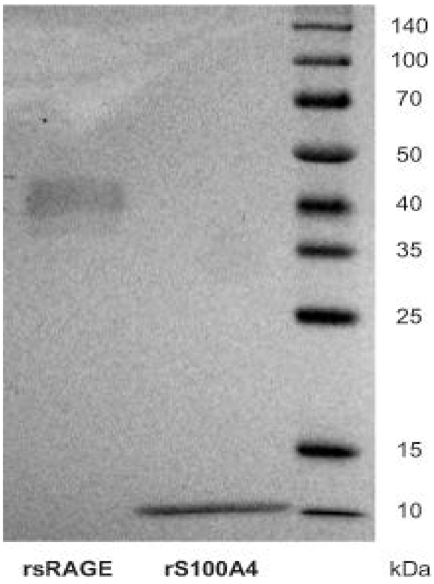

Supplementary figure: SDS-PAGE and Coomassie staining of purified rsRAGE (3 µg; lane 1) and rS100A4 (5 µg; lane 2).
